# A model for older adults’ coping with the death of their child: a grounded theory study

**DOI:** 10.1186/s12888-024-05597-3

**Published:** 2024-02-21

**Authors:** Azade Safa, Mohsen Adib-Hajbaghery, Mahboubeh Rezaei

**Affiliations:** Trauma Nursing Research Center, Kashan University of Medical Sciences, Kashan, Iran

**Keywords:** Older adults, Coping, Child’s death, Grounded theory

## Abstract

**Background:**

Losing a child at an old age while also facing health problems and physical limitations can have significant negative impacts on parents’ lives such as anxiety, depression, and impairment in social functions. The process of coping with the death of a child is particularly unknown among older adults. Therefore, this study aimed to explore how older adults cope with the death of their child.

**Method:**

This qualitative study was conducted in 2020–2021, using Corbin and Strauss (2015) approach to the grounded theory method. The sampling began purposefully and continued theoretically until theoretical saturation was achieved. Semi-structured interviews were conducted to collect data from Iranian older adults who had experienced the death of their child. To ensure data trustworthiness, the Guba and Lincoln (1985) criteria were utilized. A qualitative data analysis software, MAXQDA2020, was used to manage the data.

**Findings:**

The results of this study were obtained from 27 participants. The main concern of older adults was the fear of their lives collapsing following the death of their child. Participants utilized three main strategies to address their concerns: attempting to rebuild themselves, connecting to a higher power, and searching for positivity amidst grief. The central category that emerged from the analysis was “improving physical, mental, and spiritual capacities,” resulting in personal growth and improved social relationships.

**Conclusions:**

Through the use of the three aforementioned strategies, older adults were able to overcome their primary concern of the fear of their lives collapsing following the death of their child. Further development of the theory is suggested in order to design a model that can facilitate older adults’ coping with this difficult life event.

## Background

The death of a child is a significant crisis for older parents, with approximately 10% of them experiencing this tragedy [1, 2]. The emotional bond between parent and child deepens with age, which can lead to prolonged and intense grief with negative impacts on well-being [3, 4]. However, many parents are able to cope with and continue living without their child, which involves purposeful efforts to manage the demands created by stressful events. The capacity for coping may decrease with age and limitations. Older people are more vulnerable to the negative effects in face of child death due to the coincidence with other losses of old age such as retirement, decline in health and physical ability [5–7]. Coping with adverse conditions can lead to a better understanding of oneself and a changed perspective on life’s purpose. Parents may utilize various methods to adapt, including searching for new resources or engaging in seemingly incomprehensible behaviors [8, 9].

Cultural views of death can also influence how older parents cope with the loss of a child [10]. Culture can affect the reactions of people in different situations especially in eastern cultures. Mourning rituals are different in various cultures and religions. This ceremony can be an environment to receive parental support and release grief. Results of a study showed that the quick burial of the child with limited ceremonies caused mothers to be deprived of the support of those around them [11]. Also, another study in China showed that older adults continue their relationship with the deceased child through mourning and burial according to their culture and reach some peace [12]. In Iran children are an important source of support in old age [13], reactions to the loss of a child can have serious consequences. In a study in Iran, the use of positive religious coping mechanisms facilitated the bereavement experience [14].

Nurses are crucial in providing care for older adults in Iran, but studies suggest that mental health services may not be effective enough [15]. While nursing models for coping exist, they tend to be general and not specific to older adults [16, 17]. There is currently no available model to explain the process of coping with the death of a child and provide guidance to older adults in this context.

Previous studies on the phenomenon of child death have used quantitative research methods, but a qualitative approach was deemed more appropriate for exploring the experiences of older adults coping with the death of their child [18, 19]. The grounded theory method was utilized, which focuses on the processes and interactions underlying human phenomena [20]. The specific process of coping with the death of a child remains largely unknown, despite its significant impact on the well-being and quality of life of older adults [21]. This study was conducted to explore the process of coping with their child’s death among older adults, given their vulnerability, the lack of understanding surrounding this process, and the potential influence of cultural and ethnic differences on coping [5]. Research in this area will be useful for community policymakers and healthcare providers to help older adults cope with this crisis and guide them toward a better life.

## Methods

This study used grounded theory to explore how older adults cope with the death of their child. The study presents a final model derived from data collected between July 2020 and June 2021. A part of the study’s findings was published in previous works [22–24]. Inclusion criteria were age above 60 years old based on the definition of old age in developing countries [25], written consent to participate in the study, not having cognitive problems according to the self-report of the participants and their families, having experienced the death of his/her child (the age of the deceased child was over 16 years), and at least one year should have passed since the child’s death. The exclusion criterion was the participant’s refusal to continue the interview process.

The study used purposeful sampling, with the initial participant recommended by first researcher’s colleagues. Then, according to the data obtained from the previous interviews, the next seven participants were selected from among the eligible older adults who were registered in the primary healthcare centers of Isfahan Province. These centers are affiliated with the Ministry of Health of Iran and record the health information of all the older adults living in Iran in an integrated online system. Other participants were also introduced to the researcher in a snowball sampling by the previous participants. During a phone call, the researcher introduced himself explained the aims of the study to them, and arranged an appointment with them in case of their verbal desire to participate in this research. In the first meeting, written informed consent was obtained from the participants. Sampling continued until theoretical saturation was achieved after 31 interviews, and no new conceptual codes were identified from the data [20].

Data were collected through semi-structured, in-depth face-to-face interviews. In this study, 23 interviews were conducted at the participant’s home, three at Kashan Nursing and Midwifery College, one at the child’s grave, two at the park, and two at the health center. The reason for choosing these places was the desire of the participants and their feeling comfortable. All interviews were recorded by an MP3 recorder with the consent of the participants and researchers kept the audio files anonymous. The researcher asked preliminary questions to build trust before asking more in-depth questions about coping with the death of their child. Follow-up questions were also used to clarify responses. Due to the fatigue of some participants, interviews with four participants were conducted in two sessions. Average interview time was 45 min. Field notes were taken during the interviews. The data analysis followed the steps of Corbin and Strauss (2015), including open coding/identifying concepts, developing concepts in terms of their properties and dimensions, analyzing data for context, bringing the process into the analysis, and integrating categories [20].

For this study, data analysis involved several steps, starting with reading the interview transcripts multiple times and breaking the text down into smaller pieces. These pieces were then entered into MAXQDA2020 as in-vitro and in-vivo codes. The researcher developed concepts through comparative analysis, asking questions, and other analytical methods. Memoing was used to record any emerging ideas and relationships throughout the study. A total of 1862 primary codes were obtained from 31 interviews with 27 participants, and primary categories were identified using comparative questions related to issues that led to further conceptual development. New codes from subsequent interviews were placed in primary categories based on their similarities and differences with other codes, and each category was given a corresponding label.

Of all the participants, four had never experienced the death of their child. However, they were included based on the data collected to clarify the model. In grounded theory, the researcher may interview informed people other than the primary participants to go deeper into the data [20]. Although “having experienced the death of the child” was one of the primary inclusion criteria, the researchers interviewed some other participants based on the data obtained to complete the model. For example, one of the participants who had lost his child to COVID-19 disease said, “Because of the coronavirus situation and the fear of getting sick, no one came around us. Everyone was afraid. Even no one from the health center followed us”. The researcher therefore arranged an interview with one of the health workers at the health center to find out how these centers approached such older adults during the COVID-19 pandemic.

The study analyzed data to determine how older adults cope with the death of their child over time. The researchers identified primary categories and organized them into more abstract sub-categories, which were then grouped into main categories. They also identified underlying conditions that affected coping. To create a theoretical structure, the researchers connected sub-categories and main categories around a central category, moving concepts and categories to reveal the coping process of older adults with the death of their child (Table 1).

### Trustworthiness and rigor of the study

To ensure the data trustworthiness, the researchers used Guba and Lincoln’s criteria (1985), which included credibility, transferability, dependability, and confirmability [26]. Researcher credibility, prolonged engagement with subject matter, constant comparative analysis, and member check increased data credibility. Additionally, the researchers reviewed the data with the participants to confirm or correct the extracted codes and concepts.

The researchers used internal and external check meaning that the full text of the interviews, along with the relevant codes, concepts, and categories that emerged during the analysis, were reviewed and approved by the supervisors and advisors. The categories were also given to two colleagues outside the research team, who had a doctorate in nursing and were skilled in qualitative research, for confirmation or correction.

To increase the transferability, the researchers used maximum variation sampling and guided theoretical sampling. This involved attempting to include older people with different characteristics such as gender, age, economic and social conditions, ethnicity, and various causes of child death. The researchers also used external acceptance, where they provided the storyline and final theory of the research to four older people who experienced the death of an adult child but did not participate in the study. These individuals confirmed that the described process was largely consistent with their experiences. To increase the study dependability, the researchers used an audit trial, which involved recording a detailed description of the study process to provide conditions for the reader to follow and understand the study.

### Ethical considerations

The present research was approved by the Ethics Committee of Kashan University of Medical Sciences (code of ethics No. IR.KAUMS.NUHEPM.REC.1399.049), and written informed consent was obtained from each participant after explaining the purpose of the research. All interviews were conducted while following health protocols during the COVID-19 pandemic. Participants were assured that their audio files would be kept anonymous and confidential by the researchers, with their information recorded under a pseudonym. They were also informed that the findings would be made available to them if they wished.

**Table 1:** **A sample of the analysis process**

Main categories	Subcategories	Primary categories	Some initial coding
Achieving personal growth despite a turbulent life	Returning to the life flow	Achieving relative peace over time	-The ability to control sadness over time -Improvement of mental state over time
		Redefining the opportunity of life	-Realizing the value of life -Re-understanding the opportunity of life
	Improving social relationships	Expanding relationships to help peers	-Talking to other people who have experienced the death of a child -Giving books on the topic of death to comfort peers
		Becoming to a better person in life	-Becoming more mature in dealing with others - Taking care of people’s well-being
		Engaging in new social activities	- Helping the older adults living in care centers - Cooperate with charity foundations

## Results

This study included 27 participants, of whom 22 were older adults who experienced the death of their adult child. The age of older participants was between 61 and 85 years (Table 2).

**Table 2:** Characteristics of the study participants

Row	Gender	Age	Education level	The deceased child’s gender	Death cause	The 4 participants without experiencing their child death
1	Female	75	Diploma	Boy	Accident	-
2	Female	82	Elementary	Two girls	Kidney disease	-
3	Male	62	Diploma	Girl	Cancer	-
4	Female	63	Diploma	Girl	Cancer	-
5	Female	62	Associate degree	Boy	Burn	-
6	Female	65	Diploma	Boy	Accident	-
7	Male	63	Diploma	Boy	Martyr	-
8	Female	83	Uneducated	Boy	Overdose	-
9	Female	68	Bachelor’s	Girl	Accident	-
10	Male	61	Elementary	Boy	COVID-19	-
11	Female	50	Bachelor’s	-	-	Health Caregiver
12	Female	65	Elementary	Boy	Poisoning	-
13	Female	71	Lower secondary	Boy	Drowned	-
14	Female	35	Diploma	-	-	An older adult’s child
15	Female	65	Associate degree	Boy	COVID-19	-
16	Male	78	Uneducated	Boy	Kidney disease	-
17	Female	72	Associate degree	Girl	Burn	-
18	Female	85	Uneducated	Boy	Accident	-
19	Male	69	Bachelor’s	Boy	Accident	-
20	Female	35	PhD in nursing			-
21	Female	61	Bachelor’s	Girl	Electrocuted	-
22	Female	30	Bachelor’s	-	-	An employee in the health base
23	Male	50	Doctor	-	-	A faculty member
24	Female	65	Diploma	Boy	Murder	-
25	Female	70	Uneducated	Boy	Executed	-
26	Male	64	Bachelor’s	Girl	Suicide	-
27	Female	67	Diploma	Boy	Suicide	-

The final model was derived from 8 main categories (Table 3).

**Table 3:** Main categories and sub-categories extracted from participants’ experiences

Main categories	subcategories
Intrusion of sadness in life	- Reacting Intermittently - Fear of life collapse - Feeling sick
Double suffering following the death of a child	- Feelings of anger - Feelings of blame
Inefficiency of government support systems	- Lacking of financial support from the government - Lacking of support from healthcare system
Trying to rebuild themselves	- Distracting their minds - Practicing self-care - Keeping their child’s memory alive - Supporting other family members
Connecting to a higher power	- Relying on their personal and spiritual beliefs to cope with the loss of their child - Performing religious rites
Searching for positivity amidst grief	- Feeling that their child reached an eternal peace - Joining to realm of beauty
Supporting factors	- Self-support - Supporting from family and others
Achieving personal growth despite a turbulent life	- Returning to the life flow - Improving social relationships

### The intrusion of sadness in life

The intrusion of sadness into older adults’ lives after the death of their child can cause negative emotions and reactions that can threaten their physical and mental health. Initially, older adults experience an immediate shock and may exhibit a range of intermittent reactions such as detachment from reality, difficulty saying goodbye, restlessness, self-harm, reduced motivation to carry out daily tasks, and feelings of helplessness.

*“For the first week, I was crying all the time and did not want to eat.” (P 1)*.

After a while, they experienced a fear of life collapse. They felt that the coherence of their lives had lost cohesion and that their lives would collapse, and they feared this issue. The death of their child was a threat to the integrity of their lives, which could lead to the disintegration of family cohesion. This issue was hidden in the conversations of all the participants, some mentioned it directly, others indirectly. Other concerns that contributed to this fear were the fear of loneliness, helplessness and dependence on others, as well as caring for their spouse, other children and grandchildren.

*“All of a sudden, my whole world collapsed. I was afraid that my life would fall apart, my family would fall apart.” (P18)*.

*“After the death of my child, I became very lonely, I am afraid of helplessness and disability. My other children also suffered a lot. I am very afraid that my life will be disrupted.” (P17)*.

The stress of their child’s death and its consequences can have a significant impact on older adults’ physical and mental health and most of the participants reported suffering from some degree of chronic illness.

*“Following my son’s death, I experienced heart problems.” (P12)*.

### Double suffering following the death of a child

Many older adults in the study who lost their child due to reasons such as murder, execution, suicide, or drug abuse experienced intense emotional pain and anguish. In addition to the grief of their child’s death, they also suffered from feelings of anger and condemnation towards the person or people who caused their child’s passing.

*“Since my child’s passing, I have noticed that I get angry easily at home. I believe it is because of the intense anger I feel towards the cause of my child’s death.” (P24)*.

Many older adults blamed themselves for their child’s death and felt intense fear and anxiety about how others perceived them. The suddenness of their child’s passing made the situation even more difficult, as they had no chance to prepare for the loss. Some even wished that their child had died from a disease, as it would have given them more time to say goodbye and make peace with the situation.

*“I never thought I would witness such a devastating loss in my life. I wish she had at least gotten sick.” (P26)*.

### Inefficiency of government support systems

Many older adults cited lack of financial support from the government as a significant challenge after the death of their child. They mentioned the financial burden of holding mourning ceremonies and the lack of comprehensive insurance coverage for older people as specific areas of concern.

*“At this age, the income is not sufficient. I wish the government would give a loan for these ceremonies.” (P19)*.

From the perspective of the participants, the support of the healthcare system for older adults after the death of their child was not enough. The non-implementation of the home care program and the lack of educational and recreational programs for older adults were among these cases.

*“We became very lonely during the COVID-19 pandemic. I wish they would at least pay us a visit from the health center.” (P15)*.

### Trying to rebuild themselves

One of the key strategies older adults adopted was trying to rebuild themselves. This included distracting their minds, practicing self-care, keeping their child’s memory alive, and supporting other family members. Many older adults in the study engaged in purposeful actions after their child’s death to take care of themselves and prevent dependence on others. These actions included exercise, travel, diet modification, regular medication intake, and periodic medical monitoring.

*“I make sure to go for a walk once a day.” (P11)*.

Many older adults found that engaging in activities to keep their child’s memory alive could improve their overall health and well-being. These activities included visiting the child’s resting place, keeping their personal belongings, sharing memories with others, and observing anniversary events.

*“Since a few days before the anniversary of his passing, we have been discussing the possibility of having a grand ceremony in his honor.” (P16)*.

After the death of their child, older adults tried more to support and comfort their spouses and other children. These actions caused them to reach peace and inner satisfaction.

*“Since my son’s passing, I have been trying to spend more time with other children.” (P19)*.

### Connecting to a higher power

Many older adults in the study found that their personal and spiritual beliefs helped them cope with the loss of their child. Beliefs in a higher power gave them strength and comfort during this difficult time. Additionally, performing religious rites such as prayer and charity for their child helped them achieve peace of mind.

*“Praying helped me a lot and gave me peace.” (P21)*.

### Searching for positivity amidst grief

Many older adults in the study found that searching for a positive meaning in their child’s passing helped them cope with their grief. Some found solace in the belief that their child had achieved eternal peace and joined a realm of beauty. Others felt that their child had gone to a better world and been returned to God. All older adults tried to find more information about the world after death, and the hope of meeting their child again was a motivation to continue living.

*“After experiencing a difficult life in this world, he found relief in passing on to the next. His situation is better and he had achieved lasting peace.” (P8)*.

### Supporting factors

Supportive factors are those that protect older adults after the death of their child, and they are classified into two subcategories: self-support, support from family and others. Self-support refers to a person’s innate and relatively stable characteristics that influence their behavior. According to the participants’ perspectives, patience, realistic problem-solving, a positive attitude towards death, and learning from past experiences were helpful in coping with the loss of their child.

*“If you allow yourself to surrender to the flow of life and wait patiently, it will carry you forward.” (P6)*.

The most crucial and readily available support systems for older adults were their spouses and other children, whose support could aid them in finding peace and coping with the loss of their child.

*“My husband used to comfort me, telling me not to cry.” (P13)*.

Participants shared that they received support from those around them, including friends and neighbors following the loss of their child.

*“Every Friday, my friend sends me a bouquet of flowers, telling me that they are for my dear daughter. This small gesture brings me comfort.” (P21)*.

### Achieving personal growth despite a turbulent life

Older adults tried to achieve growth and become better individuals despite the chaos caused by the grief of losing their child. Over time, older adults could flourish and find relative peace through the strategies they used to cope with their loss.

*“Our lives will never be the same again, but… despite the chaos, I eventually found a sense of peace in my life.” (P15)*.

Older adults experienced a diverse range of social relationships following the death of their child. Through these relationships, they both gave and received support, which helped them to adapt to their new reality. As they expanded their social networks, they became more capable of helping others who had similar experiences and grew as individuals in the process.

*“Since the death of my child, I have grown more mature and strive to avoid causing others sadness.” (P3)*.

## Discussion

The study was the first qualitative research conducted in Iran that explored the coping process of older adults with the death of their child. The first main category extracted in this study was the intrusion of sadness in life. After the death of their child, the whole world of older adults had collapsed. Fear of the collapse of their lives and severe grief caused by the death of their child could threaten their health. When older adults face severe stress, emotional shock can cause a lack of control over themselves and their environment [27]. However, older adults strived to prevent their lives from collapsing as they viewed themselves as responsible for maintaining family integrity. One study showed that older adults played a vital role in strengthening family cohesion, intergenerational relationships, and caring for family members during crises [28]. Levine’s protective model emphasizes maintaining personal integrity to adapt to significant changes in life [29]. In crises, people use coping strategies to reduce their suffering [30].

The coping strategies used by older adults in this study included trying to rebuild themselves, connecting to a higher power, and searching for positivity amidst grief. Considering that Iranian people are religious, the study found that religious beliefs and engagement in religious activities helped older adults overcome feelings of sadness and despair and improve their coping [27]. A qualitative study highlighted the importance of self-management strategies among older women to control stressful conditions [31]. The present study found that older adults attempted to find positive and meaningful interpretations following their child’s death. Previous research indicated that parents experienced an identity crisis following their child’s death and attempted to find meaning to alleviate this crisis [32]. In this study, older adults who lost their child due to chronic illness viewed death as their child’s release from suffering and believed their child had reached eternal peace and joined with beauty beyond this world’s suffering. They found a sense of peace and positivity in this belief. The results of a study showed that parents considered the death of their child as a transfer to a safe place [33]. However, in previous studies, younger parents did not find any positive meaning in their child’s death [21]. These contradictions in previous research may be due to differences in age and cultural background among participants.

Some participants, in addition to the grief of their child’s death, experienced another grief, which doubled their grief. Participants who experienced unique circumstances surrounding the death of their child such as murder, execution, or suicide, experienced an additional layer of grief, resulting in feelings of anger, guilt, and fear of social stigma. Previous research has shown that the death of a loved one by suicide can evoke emotions of anger, shame, and social stigma, leaving individuals more vulnerable to the negative effects of the loss [34, 35]. Participants in this study expressed dissatisfaction with the government’s support systems, including a lack of financial support and insufficient healthcare services. In a study, older adults during the COVID-19 pandemic complained about financial problems and lack of community support [36]. The study highlighted the importance of government programs providing adequate support for older adults, particularly during crises. Participants also expressed dissatisfaction with the level of governmental support, particularly from the healthcare system, following the death of their child. They highlighted issues such as the non-implementation of home care programs and a lack of educational and recreational opportunities for older adults. A study conducted in Iran revealed that integrated and comprehensive mental health care for older adults was not effective enough [15]. Also, older adults’ home care programs have not yet been implemented in Iran [37]. Therefore, the study recommends that the healthcare system develop a follow-up program for older adults following the loss of their child, providing in-home care and monitoring these individuals’ needs and progress.

The study found that older adults used coping strategies to deal with the grief of losing their child. The result of applying these strategies was to achieve “prosperity in a turbulent life” which allowed them to return to the path of life and improve their social relationships. This aligns with previous research that has shown a direct relationship between effective coping strategies for dealing with life stress and achieving self-actualization [38, 39]. Older adults in this study engaged in new social activities, including helping their peers, after the death of their child. Social participation is a critical component of older adults’ health, and research indicates that having a robust social network and effective interactions can have a protective effect on older adults, improve their health, increase their quality of life, and prevent cognitive decline [40]. Maslow argues that facing adversity and failures can lead to self-actualization, which supports the findings of this study.

In this study, certain personality traits of older adults, including patience, and the support of family and loved ones were as supportive factors that aid in improving their physical, mental, and spiritual capacities. In Iranian culture, the family is a sacred institution and there are deep ties between family members. Older adults are respected in the family [41]. In religious recommendations and Eastern culture, older adults are under the care of the family. In this study, after the death of their child, older adults received a lot of support from their spouses and children, which helped them cope with this crisis.

### Explanation of the model

The main concern of older adults who face the death of their child is “fear of life collapse”. After the death of their child, older adults felt that the coherence of their lives would be lost and their lives would fall apart, and they were afraid of this issue. Older adults viewed the death of their child as a threat to family unity, and they strived to prevent their lives from falling apart.

The main concern of older adults following the death of their child can be attributed to two contextual factors: “double suffering” and “inefficient government support systems.” Double suffering refers to the experience of intense feelings of anger and condemnation coupled with the sudden and unexpected loss of their child. Inadequate financial support from the government and the healthcare system also contributes to older adults’ concerns about the collapse of their lives after the death of their child. The more intense these two contextual factors, the greater the fear of the collapse of life in older adults after the death of their child.

To cope with this situation, older adults employed various strategies, including trying to rebuild themselves, connecting to a higher power, and searching for positivity amidst the tragedy. The researcher conceptualized the set of these three strategies under the general strategy of “trying to improve physical, mental and spiritual capacities”. This concept indicates that older adults aim to rebuild their bodies and souls while strengthening their spiritual capacities with the help of God and their beliefs. They also sought to derive positive meaning from the heart of this tragic event to overcome their concerns and find peace.

Grief related to losing their child is an experience that casts a shadow over older adults’ lives forever, so older adults strive to maintain coherence in their lives by using the strategies mentioned above. The more they use these strategies, the more they are able to sustain this coherence. Furthermore, the application of each strategy leads to an increased ability to use other strategies effectively. Ultimately, the use of these strategies results in “prosperity in a turbulent life”. This means that older adults are able to achieve a relative sense of peace and once again find meaning in life. Through the use of these strategies, they are also able to improve their social relationships and become more perfect individuals.

**Fig. 1:**
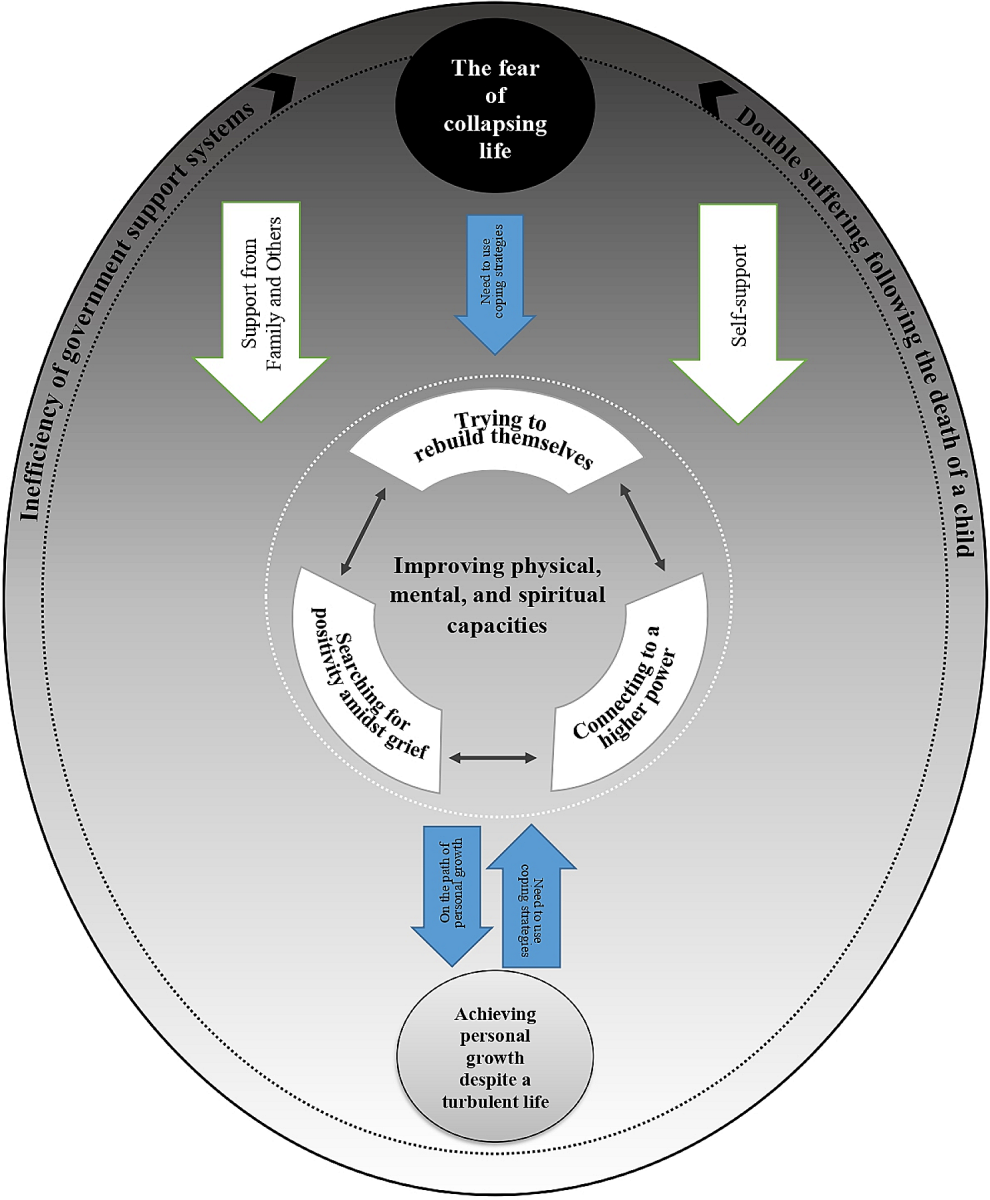
A schematic model *“*from fear of collapsing life to achieving personal growth*”*

Certain personality traits of older adults, including patience, a realistic approach to problem-solving, a positive attitude toward death, and the ability to learn from past experiences, can serve as supportive factors that aid in improving their physical, mental, and spiritual capacities. Additionally, the support of family and loved ones can help in achieving a sense of prosperity (Fig. 1).

This model can be a basis for building tools in the field of coping with the death of their child in older adults. It is also necessary to test the present model on older adults who have experienced the death of their child to determine to what extent the strategies obtained in this study can improve the coping of older adults to the death of their child.

### Limitations of the study

One of the limitations of this study was the participation of fewer fathers who experienced the death of their child. It is suggested that future studies focus more on this field. Also, considering that the majority of Iranian people’s religion was Muslim, researchers’ access to other religions was limited.

## Conclusions

The results showed that although the sadness of the death of their child has cast a shadow on the lives of older adults, they have been able to overcome their main concern, which was the fear of the collapse of their lives, by using coping strategies (trying to rebuild themselves, connecting to a higher power, and searching for positivity amidst grief). The result of using these strategies was achieving personal growth despite a turbulent life. Healthcare providers, including nurses, should understand the needs of older adults and help them adapt and cope during this crisis, and policymakers and healthcare providers should work together to assist older adults in crisis and provide the necessary conditions to guide them toward achieving coping. It is suggested that comprehensive geriatric clinics provide the necessary services including counseling for the well-being of older adults by specialists in various fields of geriatrics after the death of their child. It is also recommended to provide training in this area for healthcare team members including nurses in the form of continuous training courses.

## Data Availability

The datasets collected and analyzed in the current study are not available to the public due to ethical restrictions to protect the anonymity of participants. The corresponding author can be contacted on reasonable requests regarding the dataset.
